# Radiobioconjugate of Kadcyla with Radioactive Gold Nanoparticles for Targeted Therapy of HER2-Overexpressing Cancers

**DOI:** 10.1021/acs.molpharmaceut.5c00288

**Published:** 2025-05-30

**Authors:** Kinga Żelechowska-Matysiak, Kamil Wawrowicz, Mateusz Wierzbicki, Tadeusz Budlewski, Aleksander Bilewicz, Agnieszka Majkowska-Pilip

**Affiliations:** a Centre of Radiochemistry and Nuclear Chemistry, 86904 Institute of Nuclear Chemistry and Technology, Dorodna 16 St., Warsaw 03-195, Poland; b Department of Nanobiotechnology, Institute of Biology, 49561 Warsaw University of Life Sciences, Ciszewskiego 8 St., Warsaw 02-786, Poland; c Department of Radiology, Radiotherapy and Nuclear Medicine, National Medical Institute of the Ministry of the Interior and Administration, Wołoska 137 St., Warsaw 02-507, Poland

**Keywords:** gold-198, gold nanoparticles, T-DM1, Kadcyla, HER2+ cancer, ADC

## Abstract

One modern concept for cancer treatment is the use of antibody-drug conjugate (ADC). These therapies have shown promising results, especially in combination with other cancer treatment techniques. In this study, we propose the simultaneous use of β^−^ radiation (^198^AuNPs) and trastuzumab emtansine (T-DM1; Kadcyla) for targeted therapy of cancers with established HER2 receptor overexpression. By utilizing ^198^AuNPs-T-DM1, we aimed to reduce the required concentrations of T-DM1, which is advantageous given the associated emtansine-related side effects. In our study, we demonstrated the affinity of conjugated ^198^AuNPs-T-DM1 for HER2 receptors and its effective internalization. *In vitro* studies indicate a synergistic therapeutic effect at doses of 10 MBq/mL or 20 MBq/mL of radiation and low concentrations of Kadcyla ranging from 0.015 to 0.124 μg/mL. Treatment with ^198^AuNPs-T-DM1 at 20 MBq/mL and T-DM1 concentration of 0.031 μg/mL disintegrated 3D spheroid structures within seven days. The synthesized ^198^AuNP-T-DM1 radiobioconjugate has potential applications in nuclear medicine for treating breast or ovarian cancers with HER2 receptor overexpression.

## Graphical abstract

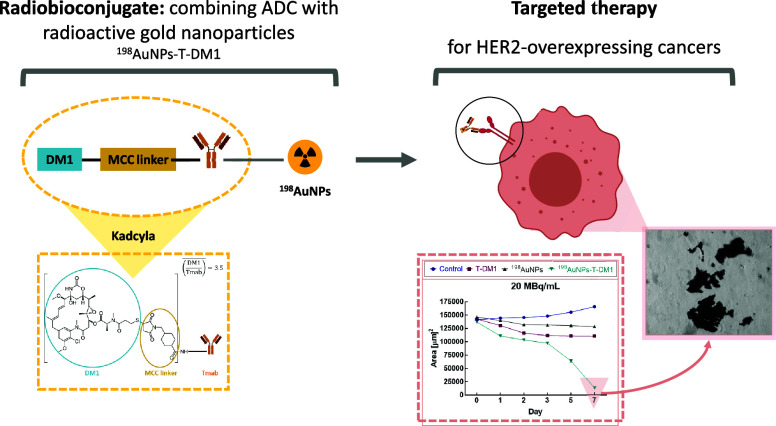

## 1 Introduction

In recent years, targeted therapy has emerged as a promising strategy for treating cancer. Unlike conventional chemotherapy and internal radiotherapy, targeted therapy delivers drugs or radiation directly to tumors, reducing the exposure of healthy tissues to these treatments.

The most common form of targeted therapy used in clinical practice is targeted chemotherapy, in which the target vector (usually a monoclonal antibody) specifically delivers a cytotoxic drug to cancer cells. The advantage of this therapy, in addition to its specificity, is the combination of immunotherapy and chemotherapy in one drug.

In the last decades, combinations of monoclonal antibodies with strong chemotherapeutic agents called antibody-drug conjugates (ADCs) have been widely used.^1^ In 2000, the United States Food and Drug Administration (FDA) approved Mylotarg for the treatment of leukemia. Mylotarg, also known as gemtuzumab ozogamicin, is a recombinant, humanized anti-CD33 monoclonal antibody (IgG4 κ antibody hP67.6) covalently attached to the cytotoxic antitumor antibiotic calicheamicin (*N*-acetyl-γ-calicheamicin) via a bifunctional linker (4-(4-acetylphenoxy)­butanoic acid).^2^ Thirteen years later, another ADC drug called Kadcyla, which targets HER2 receptors, received approval.^3^ Kadcyla (chemical name: T-DM1 or ado-trastuzumab emtansine) is a combination of the monoclonal antibody trastuzumab and the chemotherapeutic emtansine. Kadcyla was designed to deliver emtansine to HER2-positive cancer cells in a targeted way. It is currently widely used in the treatment of breast, ovarian, and stomach cancers. Recently, the FDA approved other drugs based on trastuzumab - Enhertu (fam-trastuzumab deruxtecan-nxki) for patients with unresectable or metastatic HER2 breast cancer^4^ and Tukysa (trastuzumab and capecitabine; tukatynib).^5^

In the currently most commonly used Kadcyla, each trastuzumab molecule is conjugated with approximately 3.5 DM1 molecules. After binding to the HER2 receptor, the conjugate undergoes internalization and lysosomal degradation, leading to the release of degradation products containing DM1. The trastuzumab emtansine conjugate, like trastuzumab itself, acts cytotoxically by binding to subdomain IV of the HER2 receptor, which blocks the signaling of the phosphatidylinositol 3-kinase pathway. After being released from trastuzumab, DM1 binds to tubulin, inhibiting its polymerization (binds to the tips of microtubules and thereby inhibits the growth and the shortening of microtubules, leading to suppression of microtubule dynamics), which arrests cells in the G2/M phase of the cell cycle and leads to their death by apoptosis. Thanks to this, Kadcyla delivers high doses of the drug directly to cancer cells, minimizing the exposure of healthy tissue to cytotoxic effects and reducing the side effects associated with traditional chemotherapy. Unfortunately, in many patients, especially in the advanced stages of the disease or in the case of its relapse, this drug turns out to be ineffective and causes side effects. Also, the effectiveness to the subpopulation of cancer stem cells responsible for the development and progression of the tumor is limited. Therefore, our activities focused on increasing the effectiveness and extending the action of the trastuzumab conjugate with chemotherapeutics by attaching radionuclides - emitters of corpuscular radiation.

The synergistic effect of chemo- and external radiation therapy is well-known.^6–8^ This is mainly due to the sensitizing effect of the chemotherapeutic agents for ionizing radiation. The support of chemotherapy by ionizing radiation would allow to use much lower doses of toxic chemotherapeutics.

Last year, our team worked on developing bioconjugates that combine chemotherapeutics and radionuclides in one drug. We have utilized nanoparticle systems, such as gold nanoparticles and liquid crystal cubosomes, as platforms for drug delivery. Our research has been focused on studying conjugates of doxorubicin and radionuclides ^177^Lu, ^213^Bi, and ^198^Au immobilized together on nanostructures.^9,10^ In vitro, experiments have shown that the combined radiobioconjugates exhibit a synergistic effect compared to using the two therapeutic methods separately. However, we have observed that in our systems, the chemotherapeutic agent was not released in the cytoplasm, which reduced its cytotoxicity.

Therefore, in our future work, we have chosen to use the already mentioned Kadcyla conjugate along with radionuclides that emit β^−^, α, and Auger radiation. This system will help in selectively delivering and releasing the chemotherapeutic drug DM1 in cancer cells, in addition to providing internal radiotherapy.

In our first presented studies, Kadcyla was conjugated to radioactive gold nanoparticles. Such a system will target the HER2 receptor and have an immunotherapeutic and chemotoxic effect. Additionally, the attached ^198^Au radionuclide will ensure the radiotoxicity of the bioconjugate.

^198^Au is a β^−^ emitter with a β_max_ energy of 0.96 MeV and a half-life of 2.7 days.^11,12^ It can be obtained with very high activity by thermal neutron radiation of the monoisotopic ^197^Au target. The high cross-section for ^197^Au­(n,γ)^198^Au nuclear reaction (98.7 barns) allows to receive 350 GBq of ^198^Au (1 mg Au target, 1.5 × 10^15^ n/cm^2^/s neutron flux, 70 h irradiation). After irradiation under these conditions, about 5% of gold atoms are radioactive. However, due to the high molecular mass of trastuzumab, this results in a low specific activity of the radioconjugate, leading to low cytotoxicity. As it has been found that trastuzumab can be labeled with chelator molecules to attach up to only 6 radionuclide atoms.^13^

To increase the specific activity of the radioconjugate, we decided to attach ^198^Au in the form of a radioactive nanoparticle. This approach allows for the attachment of 40,000 radioactive ^198^Au atoms when using 30 nm Au nanoparticles, significantly increasing specific activity.

Another advantage of presented combined therapy is that chemotherapy drugs need to reach and enter cancer cells to work, while with radionuclide therapy using β^−^ radiation, the radiolabeled compound does not necessarily need to cross the tumor cell to be effective. The emitted particles can interact with cells near the target, not just the ones with the targeted epitope. This “crossfire effect” is important for treating tumors with different antigen or receptor expression or with poor blood supply.^14^

The goal of our studies was to develop a new drug that combines the immunotherapeutic and chemotherapeutic properties of Kadcyla with the radiotoxic effects of β^−^ radiation emitted by ^198^Au. We also aimed to evaluate how the combination of these modalities would impact the treatment of HER2-positive tumors.

## 2 Material and Methods

### 2.1 Reagents

#### Chemical Reagents

Millipore Sigma (St. Louis, MO, USA): gold­(III) chloride trihydrate (HAuCl_4_·3H_2_O), sodium hydroxide (NaOH), trisodium citrate dihydrate (C_6_H_9_Na_3_O_9_) and poly­(ethylene glycol) (HS-PEG-COOH, 5 kDa); Creative PEGworks (Chapel Hill, NC, USA): alpha-pyridyl-2-disulfid-omega-carboxy succinimidyl ester poly­(ethylene glycol) (OPSS-PEG-NHS, 5 kDa); Chempur (Piekary Śląskie, Poland): hydrochloric acid (HCl, 35−38%) and nitric acid (V) (HNO_3_, 65%); Thermo Fischer Scientific (Waltham, MA, USA): iodogen (1,3,4,6-tetrachloro-3R,6R-diphenylglycouril); GE Healthcare (Piscataway, NJ, USA): PD-10 column; Polatom (Otwock-Świerk, Poland): saline solution (NaCl); natural gold metallic target (99.99%). T-DM1 was isolated from Kadcyla (Roche Pharmaceuticals, Basel, Switzerland) and trastuzumab from Herceptin (Roche Pharmaceuticals). Ultrapure deionized water (18.2 MΩ·cm; Hydrolab, Straszyn, Poland) was used to prepare the aqueous solutions.

#### Biological Reagents

Biological Industries (Beth Haemek, Israel): McCoy’s 5A, Dulbecco’s modified eagle medium (DMEM), penicillin and streptomycin, heat-inactivated fetal bovine serum, trypsin EDTA solution C, and phosphate-buffered saline (PBS); Promega (Mannheim, Germany): CellTiter96 Aqueous One Solution Reagent (MTS compound) and dimethyl sulfoxide (DMSO); BD Biosciences (Becton, Dickinson and Company, New Jersey, USA): FITC Annexin V, Propidium Iodide Staining Solution (PI) and 10X Annexin V Binding Buffer, and RNase; ChemPur (Piekary Śląskie, Poland): ethanol and Tween 20; Thermo Fischer Scientific (Waltham, MA, USA): Hoechst 33258 and Triton X-100 Surfact-Amps Detergent Solution; Millipore Sigma (St. Louis, MO, USA): Anti-Human IgG, F­(ab′)­2 fragment−FITC antibody produced in goat, paraformaldehyde (PFA), and bovine serum albumin (BSA); Agilent Technologies (Santa Clara, CA, USA): DAKO Fluorescent Mounting Medium.

The cell lines (SKOV-3, MDA-MB-231) used were cultured according to the American Type Culture Collection protocol (ATCC, Rockville, MD, USA).

### 2.2 Radionuclides

For radioiodination of T-DM1, noncarrier-added ^131^I was used. The radioisotope in the form Na^131^I; with specific activity >550 GBq/mg) was obtained from the POLATOM Radioisotope Centre (Świerk, Polska). The MARIA research reactor at the NCBJ (National Centre for Nuclear Research; Świerk, Poland) was used to obtain gold-198 radionuclide. A solid target of gold-197 was irradiated by neutron flux 1.5 × 10^14^ n s^−1^ cm^−2^ and then cooled for 12 h. The details of target preparation and the target dissolution have already been described earlier.^9^

### 2.3 HR-TEM Method

The size and shape of the 30 nm AuNPs were confirmed by HR-TEM microscopy (TALOS F200X, Thermo Fischer Scientific-Waltham, MA, USA). Dynamic light scattering (DLS) (Zetasizer Nano ZS DLS, Malvern, UK) was used to determine the size, polydispersity index, and zeta potential. The stability test was performed under the same conditions as described.^15^

### 2.4 Synthesis of AuNPs-T-DM1/^198^AuNPs-T-DM1

The synthesis of 30 nm AuNPs nanoparticles as well as radioactive gold nanoparticles ^198^AuNPs was performed as described.^10,16^ Briefly, for T-DM1 conjugation (200 μg), a 25-molar excess of OPSS-PEG-NHS (5 kDa) in sodium carbonate buffer (pH ∼8.90, 100 mM) was used. Synthesis was carried out overnight at room temperature. Vivaspin500 100 kDa cutoff centrifuge concentrators (Sartorius, Goettingen, Germany) were used to purify free OPSS-PEG-NHS. Then, T-DM1 was added to AuNPs/^198^AuNPs (Table 1), and the synthesis was carried out for 30 min at room temperature. To remove excess unconjugated protein, samples were subjected to centrifugation (10 min, 13 000 rpm). In the next step, 15000-molar excess of HS-PEG-COOH (5 kDa) was conjugated for 30 min and centrifuged again (10 min, 13 000 rpm). AuNPs-T-DM1/^198^AuNPs-T-DM1 were dispersed in 100−1000 μL of deionized water.

### 2.5 Binding Studies

SKOV-3 and MDA-MB-231 cells (6 × 10^5^) were seeded into 6-well plates (TPP, Switzerland) and kept in an incubator for 24 h before the experiment. The wells were rinsed with PBS before the addition of ^198^AuNPs-T-DM1. The compound was incubated at different concentrations for 1.5 h in the incubator. After the medium was collected, PBS was again used to wash the cells. One M NaOH was utilized to collect the cell fractions. A Wizard^2^ Detector Gamma Counter (PerkinElmer, Waltham, MA, USA) was applied to measure media and cell activity. To determine specific binding, the ratio of total to nonspecific binding was determined. A 100-mol excess of unconjugated trastuzumab was used to inhibit HER2 receptors.

### 2.6 Internalization Studies

Similar to the binding studies, cells were prepared for the internalization experiment. Then,

Five nM bioconjugate was added to the medium and incubated at 4 °C for 1 h after removal of the medium. The fraction was collected into vials and the cells then got two washes with PBS before receiving 1 mL of new cell medium. Plates were incubated for 1, 6, 18, and 24 h. The medium was then removed and collected, and the cells were washed two times with PBS, twice (2 × 5 min in the fridge) with 0.05 M glycine·HCl pH = 2.8 and twice with 1 M NaOH to harvest the cells. Unconjugated trastuzumab in excess of 100 mol was used to block HER2 receptors. Media and cellular activity were measured using a Wizard^2^ Detector Gamma Counter.

### 2.7 Confocal Microscopy Imaging

Five 12 mm diameter sterile coverslips (Thermo Fischer Scientific, Waltham, MA, USA) were used to seed SKOV-3 and MDA-MB-231 cells in six-well plates (2 × 10^5^ cells per well), and the cells were then left to incubate overnight. Compounds were then added, the medium removed, and incubated for 24 h. Afterward, 24-well plates were prepared with 1 mL of PBS per well. Coverslips were transferred to the wells (1 coverslip per well). A 4% PFA solution (for fixation) was added for about 10 min, after which the wells were rinsed twice with PBS. Then 0.1% Triton X-100 (for permeabilization) was used and after 5 min, the wells were rinsed with PBS. For a 30 min incubation, 5% BSA in TBST was added. The wells were then washed with TBST solution (3 × 5 min). Anti-Human IgG was then added and incubated for 1 h in the dark and then washed again with TBST (3 × 5 min). In the next step, Hoechst 33258 was added and the plates were left in the incubator for 15 min. After cleaning the wells three times with TBST solution (using DAKO), the coverslips were transferred to the primary slides with special care. Slides were dried and stored at 4 °C in the dark. For imaging with Hoechst 33258 staining, wavelengths of 352 and 461 nm were used, for Anti-Human IgG staining - wavelengths 491 and 516 nm. An FV-1000 confocal microscope (Olympus Corporation, Tokyo, Japan) was applied for the study, and FV10-ASW 4.02 software (Olympus Corporation) and Fiji (Fiji Is Just ImageJ)^16^ were used for image analysis.

### 2.8 Cytotoxicity Studies (MTS)

Cytotoxicity studies were performed on two cell lines using different concentrations of T-DM1, Trastuzumab, ^198^AuNPs, and ^198^AuNPs-T-DM1. Cells were prepared 24 h before the experiment (seeding: SKOV-3 2.5 × 10^3^ cells, MDA-MB-231 2 × 10^3^ per well) and incubated at 37 °C in an atmosphere of 5% CO_2_. After removal of the medium, tested compounds were added and incubated for 24, 48, and 72 h. After incubation, the wells were washed with PBS, a new medium was added, and then 20 μL of MTS. Absorbance at 490 nm was measured using a microplate reader.

### 2.9 Flow Cytometry: Apoptosis and Cell Cycle Assay

SKOV-3 cells were seeded in 6-well plates (6 × 10^5^ per well) for the apoptosis assay. After overnight incubation, compounds were added and incubated for 24 and 48 h. 1X Annexin V Binding Buffer, 5 μL FITC, and 5 μL PI were added to the collected cells (after trypsin and PBS). Incubation duration was 15 min at 37 °C. Samples were analyzed on a FACSCelestaTM instrument (BD Biosciences, San Jose, CA, USA) using FACSDivaTM v8.0 software (BD Biosciences, San Jose, CA, USA).

Cells in the cell cycle assay were subjected to the same procedure. However, after using PBS, cells were suspended in 70% ethanol and frozen for 1.5−2 h. Ethanol was then removed from the samples and washed with PBS. Then 20 μL of PI with 2 μL of RNase was added.

### 2.10 Spheroids

SKOV-3 cells were seeded in 96-well plates with an ultralow adhesion surface (Corning, NY, USA) and cultured for 5 days, changing the medium every 2 days. After this time, the test compounds were added, and spheroids were measured for 1 week (the medium was still changed every 2 days). A Primovert Color Axiocam 305 microscope (Zeiss, Jena, Germany) with ZEN 3.0 lite software (Zeiss, Jena, Germany) was used for imaging and data processing.

### 2.11 Statistical Analysis

GraphPad Prism software version 8.0 (GraphPad Software Inc., San Diego, CA, USA) was used to perform the statistical analysis of the experimental data. Student’s *t* test and one-way ANOVA were performed and parameters obtained were considered statistically significant when *p* ≤ 0.05 (*), *p* ≤ 0.01 (**), *p* ≤ 0.001 (***), and *p* ≤ 0.0001 (****).

## 3 Result and Discussion

### 3.1 Synthesis and Characterization of AuNPs-T-DM1/^198^AuNPs-T-DM1

Scheme 1 illustrates the synthesis process of the ^198^AuNPs-T-DM1 radiobioconjugate. As presented below, Kadcyla consists of a trastuzumab antibody linked to the potent emtansine-DM1 (a derivative of maytansine) by a noncleavable MCC (maleimidomethyl cyclohexane-1-carboxylate) linker. The conjugate OPSS-PEG-NH-T-DM1 was synthesized by attaching OPSS-PEG-NHS to the amino group of the lysine in trastuzumab. This compound was then conjugated to ^198^AuNPs through a strong gold−thiol bond. Next, the nanoparticles were centrifuged, and additional HS-PEG-COOH was added to prevent agglomeration. Finally, the synthesis was subjected to another centrifugation and dispersed as described previously.^9^

**Scheme 1:**
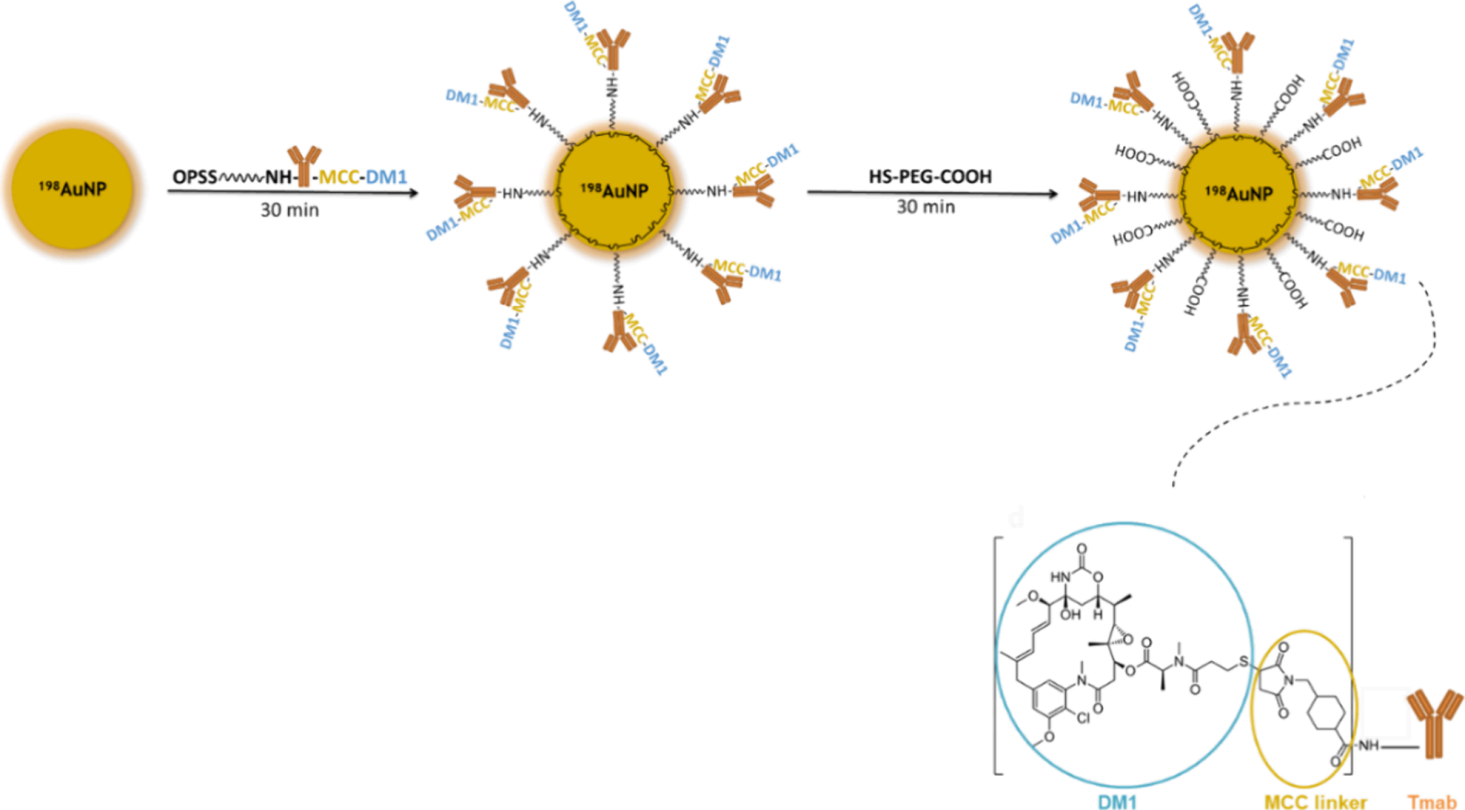
Scheme for the Synthesis of ^198^AuNPs-T-DM1 Radiobioconjugate

HR-TEM images^15^ proved the sphericity of the nanoparticles and their size of 30 nm. The hydrodynamic diameter, polydispersity index (PDI), and zeta potential of AuNPs-T-DM1 were tested by DLS. The data presented in Table 1 indicate that the hydrodynamic diameter values increased after each stage, confirming the surface modification of AuNPs. The attachment of T-DM1 increased the size from 34.72 nm ± 0.22 to 57.75 nm ± 0.61, as shown in the table. Additionally, the PDI was less than 0.2,^17^ and the high negative zeta potential values indicated no tendency for agglomeration.

**Table 1:** DLS Results of Hydrodynamic Diameter and Zeta Potential Analysis

**compound**	**hydrodynamic diameter [nm]**	**polydispersity Index (PDI)**	**zeta potential [mV]**
AuNPs	34.72 ± 0.22	0.161 ± 0.002	−45.3 ± 3.5
AuNPs-T-DM1	57.75 ± 0.61	0.124 ± 0.005	−32.3 ± 1.7

Moreover, the results of the colloidal stability tests presented in Figure 1 confirmed the previous conclusions about the lack of agglomeration tendency. The AuNPs-T-DM1 bioconjugate was stable for 1 week in the tested media (NaCl, PBS). Unfortunately, the DLS method cannot be used for stability testing in human serum (HS) or culture medium containing fetal bovine serum (FBS) due to the high protein content. The results we obtained are consistent with those from our previous work,^15^ where trastuzumab and a chemotherapeutic agent were also attached to AuNPs. The conjugate was stable for up to 7 days. The same stability was also achieved in a study where trastuzumab was conjugated to platinum-coated gold nanoparticles (Au@Pt-PEG-trastuzumab). The obtained results show that the bioconjugate maintains colloidal stability for more than two half-lives of ^198^Au (*t*_1/2_ = 2.7 d), ensuring effective therapy.

**Figure 1:**
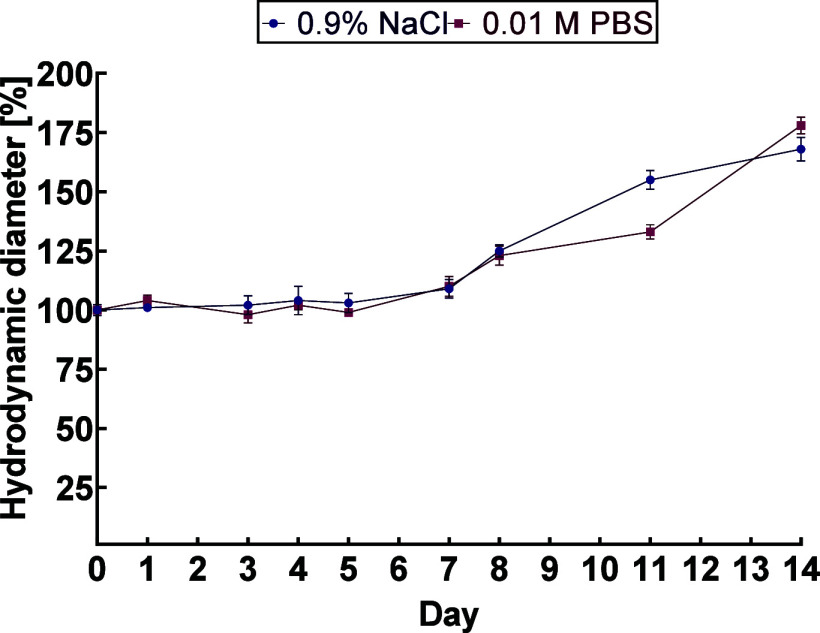
Hydrodynamic diameter changes of AuNPs-T-DM1 (incubated in two media: 0.9% NaCl and 0.01 M PBS) at 37 °C as a function of time.

### 3.2 Binding and Internalization Studies

To test the compound’s affinity for HER2 receptors, a ^198^AuNPs-T-DM1 binding assay was performed on two cancer cell lines, SKOV-3 and MDA-MB-231. Radiobioconjugate binding was tested on blocked (after prior addition of trastuzumab) and unblocked cells. Results indicating specific binding to the receptor were obtained on the HER-positive line, as presented in Figure 2A. The blue points represent the total results, encompassing both specific and nonspecific binding. The green points indicate the values for specific binding, while the orange points correspond to nonspecific binding. The results showed high receptor affinity of synthesized radiobioconjugate to HER2 receptors overexpressed on SKOV-3 cells.

Additionally, a test for internalization was conducted on both cell lines. The results showed that the internalized fraction of the radiobioconjugate was more than 97% (97.0 ± 4.0) after 1 h and remained at a similar level, slightly increasing to 99.21 ± 0.30 after 24 h (Figure 2B). Furthermore, the results from Figure 2C, presenting the percentage specificity of the compounds, show statistical differences between the HER2+ line (SKOV-3) and the HER2 negative line (MDA-MB-231) at the first two measurement points (1 h, 6 h). The values obtained are consistent with those found in other works where binding and internalization of AuNPs-trastuzumab were investigated.^9,15,18,19^

**Figure 2:**
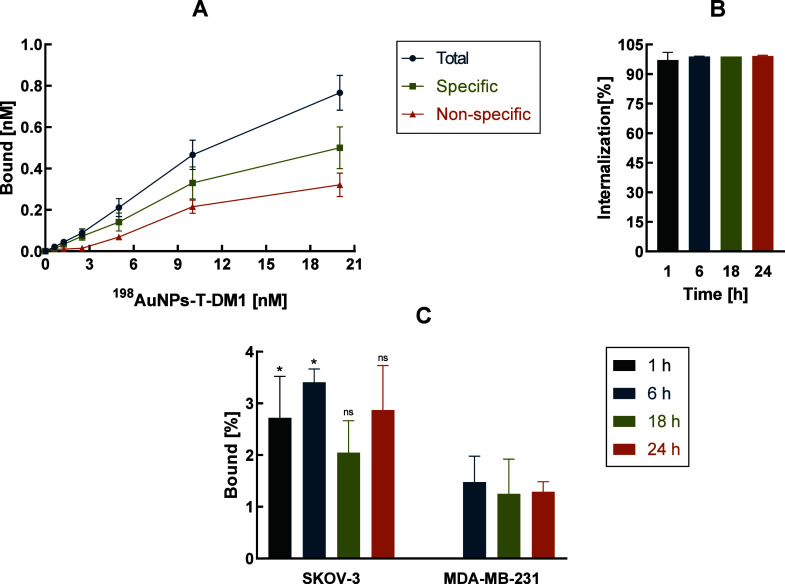
Specificity of binding ^198^AuNPs-T-DM1 on SKOV-3 (HER2+) (A). Percentage bioconjugate internalization results obtained for SKOV-3 (HER2+) (B). Percentage bioconjugate specificity results achieved for SKOV-3 (HER2+) and MDA-MB-231 (HER2-) cell lines (Student’s *t* test, *p ≤ 0.05, ns = nonsignificant) (C).

Furthermore, the results of internalization studies using the ^198^Au radiotracer were supported by confocal imaging, presented in Figures 3 and 4. Figure 3 shows images of SKOV-3 cells treated with AuNPs-PEG-COOH (column B), T-DM1 (column C) and AuNPs-T-DM1 (column D). The pictures obtained for cells treated with AuNPs-PEG-COOH are similar to those of control cells, where only Hoechst 33528 staining (blue fluorescence) is visible. Acquired confocal images clearly indicated that only trastuzumab-conjugated NPs are able to locate inside HER2+ cancer cells. As shown, PEG-ylated NPs (Au-PEG-COOH) were not detected inside the SKOV-3 cells, thus revealing the fundamental role of trastuzumab as a targeting vector. Merged signals demonstrated the successful penetration of the bioconjugate particles into the SKOV-3 cells and precise localization nearly to the nuclear envelope area. The attachment of AuNPs to T-DM1 reduces its internalization, but its localization in the cytoplasm is still observed. Hence, the accumulation of AuNPs-T-DM1 in close proximity to the most sensitive cellular organelle led us to anticipate high cytotoxicity induced by bioconjugates. The localization of T-DM1 and AuNPs-T-DM1 inside the cell nucleus was not observed.

Figure 4 presents comparative images of the bioconjugate for the SKOV-3 and MDA-MB-231 cell lines. The green signal, originating from trastuzumab-DM1, is not observed in the receptor-negative cell line, indicating the lack of specific binding and further entry into cells. In contrast, for the HER2+ cell line, the internalization of AuNPs-T-DM1 in cytoplasm is observed. The obtained images confirm that the presence of HER2 receptors is necessary for the internalization of T-DM1 and AuNPs-T-DM1. According to the literature,^1^ Kadcyla is internalized via endocytosis after binding to the HER2 receptor. Subsequently, endocytic vesicles through the endosomal pathway are matured for delivery to lysosomes and then DM1-containing catabolites are released into the cytoplasm through lysosomal degradation. Thus, tubulin polymerization is inhibited, ultimately leading to cell death.^20–24^ Results obtained by the radiometric assay, together with the results presented in Figures 3 and 4, show the internalization of AuNPs-T-DM1 into the cytoplasm in a manner similar to T-DM1, although to a lesser extent. This enables lysosomal release of the chemotherapeutic DM1 and leads to the mechanism of action described above. These findings are consistent with predictions and align with other studies where nanoparticles have been examined in conjunction with trastuzumab.^15,18,25–27^

**Figure 3:**
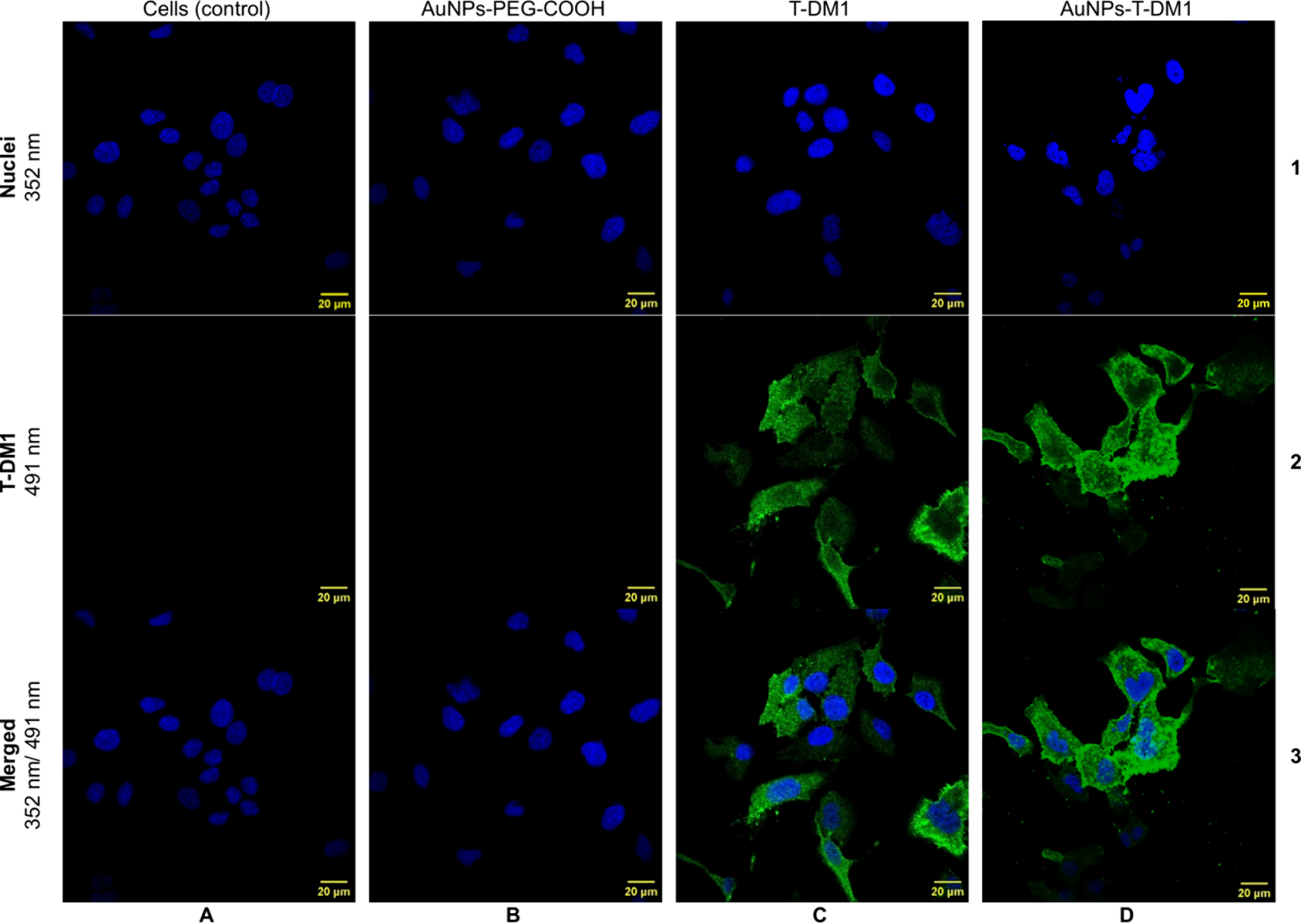
Confocal microscopy images of SKOV-3 cells treated with AuNPs-PEG-COOH (column B), T-DM1 (column C), and AuNPs-T-DM1 (column D). The first column (A) is the control (untreated) cells. Fluorescence signals used: blue = cell nuclei, Hoechst (row 1) and green = trastuzumab (T-DM1), FITC (row 2).

**Figure 4:**
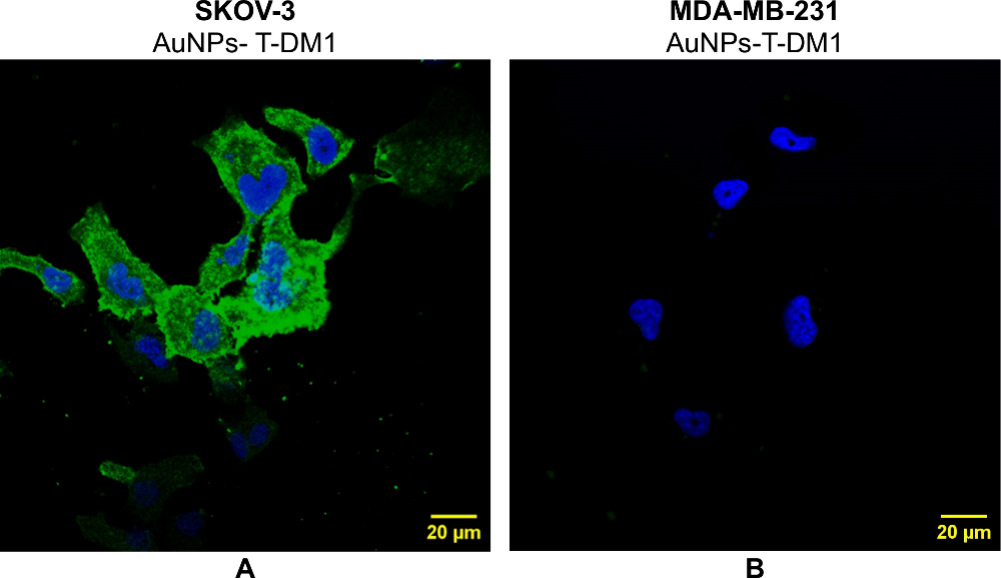
Confocal microscopy images of SKOV-3 and MDA-MB-231 cells treated with AuNPs-T-DM1. Fluorescence signals used: blue = cell nuclei, Hoechst and green = trastuzumab (T-DM1), FITC.

### 3.3 MTS Assay

The viability of SKOV-3 cells incubated with various concentrations of trastuzumab and T-DM1 was evaluated using the MTS assay. The aim of these studies was to investigate whether trastuzumab and T-DM1 can lead to mitochondrial dysfunction and cell death. In Figure 5, the cell viability in different concentrations of trastuzumab, and T-DM1 is presented at three-time points: 24, 48, and 72 h. Even at the highest concentrations tested, trastuzumab demonstrated no toxic effects on the SKOV-3 cell line. In contrast, the lowest used concentration of T-DM1 (2.5 μg/mL) resulted in 53.7 ± 2.7% live cells after 72 h. The study confirmed that T-DM1 exhibited significantly stronger toxicity compared to trastuzumab alone. A similar effect was observed in another experiment involving SKOV-3 cells, where this cancer line demonstrated inhibition of cell growth after treatment with T-DM1 but exhibited no response to unconjugated trastuzumab.^22^ Other studies have noted that the cellular efficacy of T-DM1 is not necessarily correlated with the sensitivity of cells to trastuzumab.^28^

**Figure 5:**
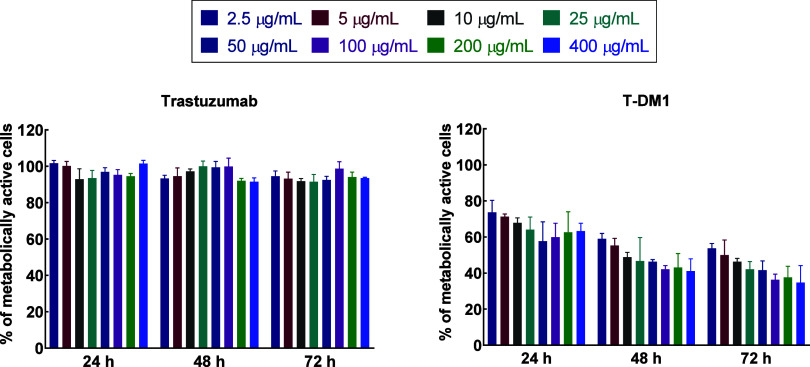
Comparison of the metabolic activity of SKOV-3 cells after treatment with different concentrations of trastuzumab and T-DM1 after 24, 48, and 72 h. Untreated cells were used as a control for 100% viability.

The aim of the studies was to enhance the cytotoxicity of T-DM1 by combining the chemotoxicity of DM1 with the radiotoxic effect of β^−^ radiation emitted by ^198^Au. To observe the radiotoxic effect of the radiobioconjugate, low concentrations of T-DM1 ranging from 0.015 to 0.124 μg/mL were tested. The highest concentration of T-DM1 tested in the experiment, 0.124 μg/mL, resulted in 58 ± 13% viable cells after 72 h. Other research groups have investigated the impact of T-DM1 on the SKOV-3 cell line, producing inconsistent results. These variations can be attributed to differences in methodologies, especially regarding the number of cells used per well. Reported IC_50_ values after 72 h included 0.009 μg/mL,^22^ 0.0225 μg/mL,^29^ and 1.2 μg/mL.^28^

Based on the outcomes achieved for T-DM1 (Figure 5), cytotoxicity testing of radioactive ^198^AuNPs and ^198^AuNPs-T-DM1 was performed, and the results are presented in Figure 6 for HER2 positive SKOV-3 cell line and in Figure 7 for HER2 negative MDA-MB-231 cell line. As shown, the decrease in metabolic activity depends on incubation time, radiation dose (doses tested -2.5, 10, and 20 MBq/mL), and T-DM1 concentrations. Lower cytotoxicity of ^198^AuNPs on SKOV-3 cells and ^198^AuNPs-T-DM1 on MDA-MB-231 cells indicate that due to the lack of internalization via HER2 receptors, the chemotoxic effect of TDM1 is negligible. In this case, the observed cytotoxic effect is related only to the radiotoxicity of β^−^ radiation emitted by ^198^Au. Its range is so long that its cytotoxicity does not require conjugate internalization. Similar effects were previously observed when studying the impact of the ^198^AuNP-T-Doxorubicin conjugate on the SKOV-3 and MDA-MB-231 cell lines.^9^

The analysis of the results from the MTS tests shows that combining radiotherapy with the chemotherapeutic effect of ADCs resulted in a synergistic effect (*I* > 0)^30^ for all concentrations of T-DM1 tested at a dose of 20 MBq/mL. At a dose of 10 MBq/mL, synergistic effects of the combination therapy were observed after 24 h for all used concentrations. The lowest dose tested, 2.5 MBq/mL, when combined with the highest concentration of Kadcyla (0.062 μg/mL after 48 and 72 h), resulted in greater toxicity at all time points compared to separate uses of radiation and T-DM1. These results show that combination therapy with radiation (especially at 20 MBq/mL) and ADCs is more effective than formerly proposed therapy with doxorubicin combined with ^198^Au.^9^ Previous work demonstrated only a synergistic effect in 3D cell cultures (spheroids). However, in the MTS assay, the differences between radioactive nanoparticles with attached chemotherapeutic and radiobioconjugate were statistically insignificant. The crucial point in effective therapy using ADC is the release of a chemotherapeutic agent inside the cell, as is the case with T-DM1.^21,31^

**Figure 6:**
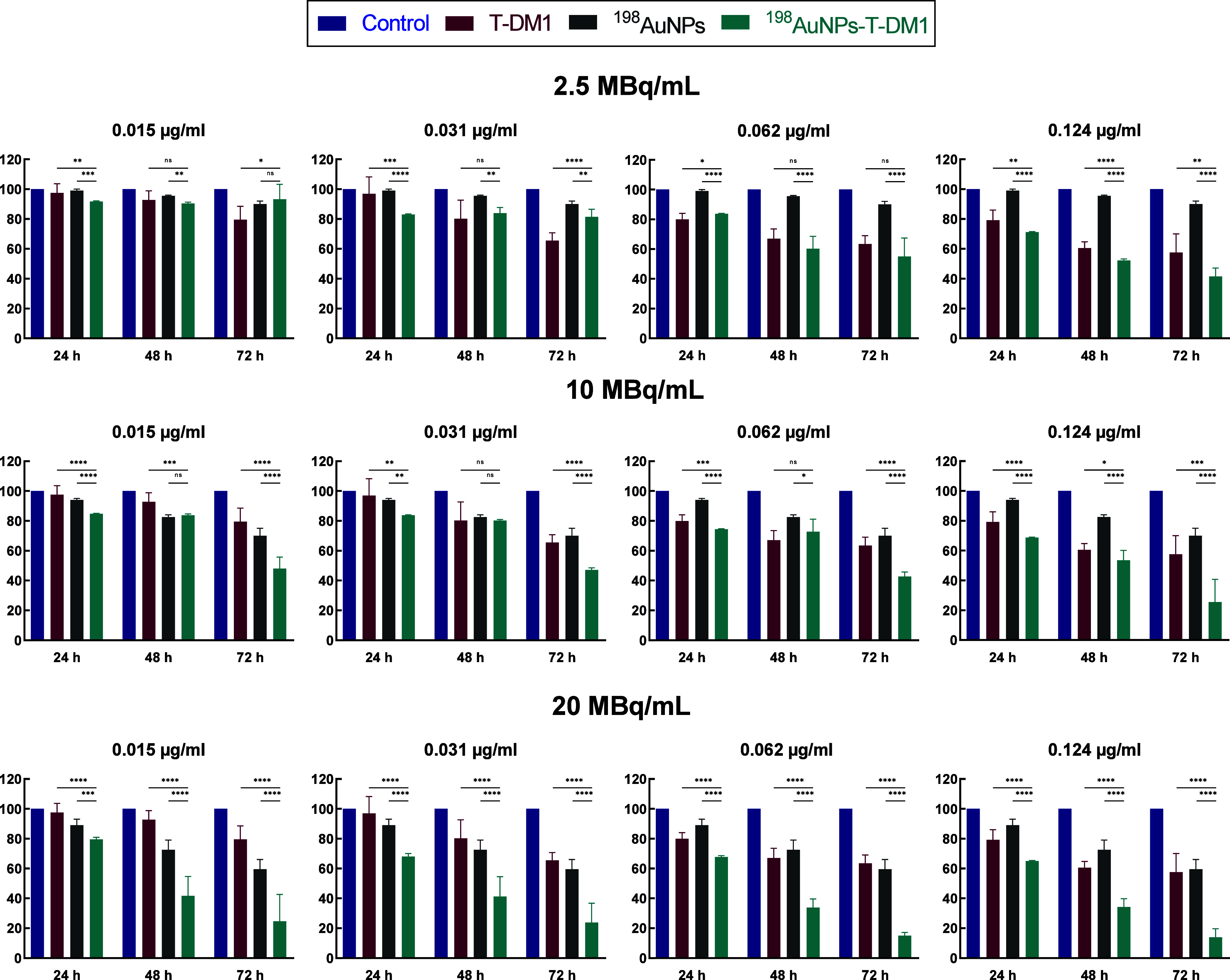
Summary of metabolic activity of SKOV-3 (HER2+) cells treated with various activity doses (2.5, 10, and 20 MBq/mL) and concentrations of ^198^AuNPs-T-DM1 (0.015, 0.031, 0.062, and 0.124 μg/mL) after 24, 48, and 72 h. Untreated cells were used as control (100% viability). A one-way ANOVA test was used for statistical analysis; statistical significance was tested for T-DM1 vs ^198^AuNPs-T-DM1 and ^198^AuNPs vs ^198^AuNPs-T-DM1 groups. A *p*-value, *p* ≤ 0.05, was considered statistically significant (*), *p* ≤ 0.01 (**), *p* ≤ 0.001 (***), and *p* ≤ 0.0001 (****); nonsignificant (ns); mean ± SD, *n* = 3.

**Figure 7:**
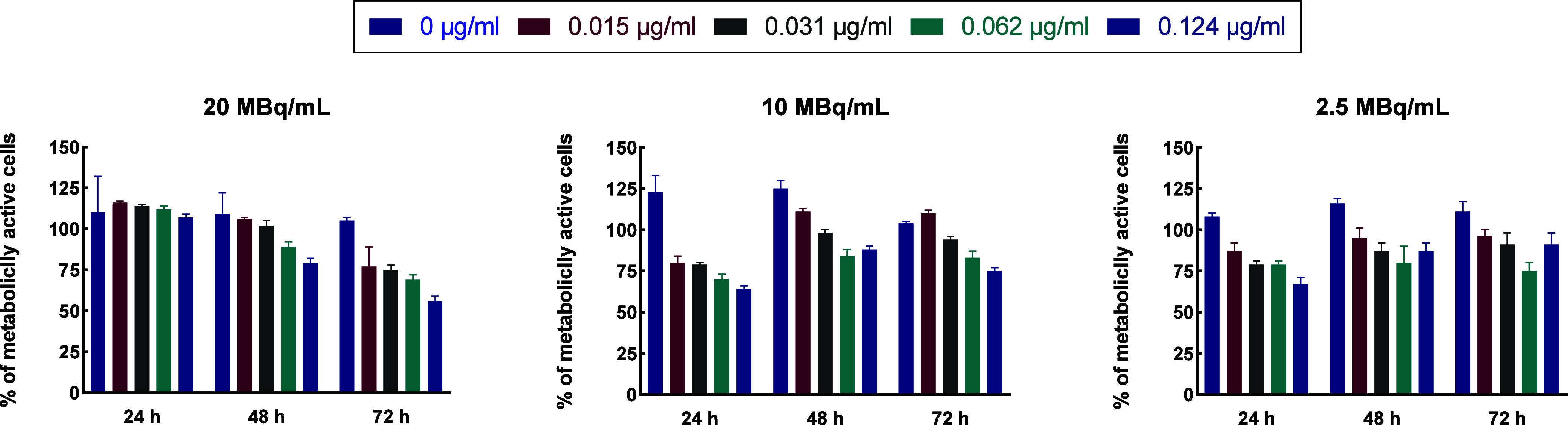
Summary of metabolic activity of MDA-MB-231 (HER2-) cells treated with different activity doses (2.5, 10, and 20 MBq/mL) and concentrations of ^198^AuNPs-T-DM1 (0.015, 0.031, 0.062, and 0.124 μg/mL) after 24, 48, and 72 h. Untreated cells were used as control (100% viability). A one-way ANOVA test was used for statistical analysis; statistical significance was tested for T-DM1 vs ^198^AuNPs-T-DM1 and ^198^AuNPs vs ^198^AuNPs-T-DM1 groups. A *p-*value, *p* ≤ 0.05, was considered statistically significant (*), *p* ≤ 0.01 (**), *p* ≤ 0.001 (***), and *p* ≤ 0.0001 (****); mean ± SD, *n* = 3.

### 3.4 Apoptosis

Based on the MTS results, apoptosis analysis was performed using a single concentration of Kadcyla -0.031 μg/mL (Figure 8). Some studies confirm that T-DM1 induces apoptosis^22^ and autophagy.^22,32,33^ Therefore, the expected outcome with the combination therapy of Kadcyla and ionizing radiation is that most cells will undergo apoptosis (both early and late stages). In the cytotoxicity test conducted by Lewis et al., it was shown that the IC_50_ for the BT-474 cell line (0.004 μg/mL) was more than twice as low as for the SKOV-3 cell line (0.009 μg/mL).^22^ It is interesting to note that in the apoptosis experiments conducted after 48 h for the BT-474 cell line using a dose significantly higher -0.5 μg/mL, compared to that used in our studies, (0.031 μg/mL), cell death mainly occurred through early apoptosis (20.22%), with late apoptosis accounting for 9.5% of the cells. In our experiments, where of SKOV-3 cells were treated with T-DM1, the majority of cells died due to late apoptosis (13.2 ± 1.7%), with a small percentage undergoing early apoptosis (3.46 ± 0.46%). Despite SKOV-3 cells being more resistant to Kadcyla compared to the BT-474 cell line, this study achieved a comparable effect with combined therapy (^198^AuNPs-T-DM1, dose of 20 MBq/mL) as in Lewis et al.’s work using T-DM1, but at over 16 times lower concentration of Kadcyla.^22^ In the cited article, it was reported that 27.92% of BT-474 cells treated with T-DM1 died due to apoptosis after 48 h, whereas in this study, a higher result was achieved -31.8% of cells underwent apoptosis. The findings after 48 h are consistent with the results obtained from the MTS cytotoxicity assay. Considering the percentage of late apoptosis in untreated cells (9.7 ± 0.3%), it is evident that the effect of ^198^AuNPs-T-DM1 (27.1 ± 4.4%) was stronger than the combined effects of T-DM1 (13.2 ± 1.7%) and ^198^AuNPs (14.20 ± 0.51%). Results from the Annexin V assay analysis indicate statistically significant differences between T-DM1 vs ^198^AuNPs-T-DM1 and ^198^AuNPs vs ^198^AuNPs-T-DM1 supporting the conclusion of a synergistic effect after 48 h as observed in the cytotoxicity test (MTS) previously described.

**Figure 8:**
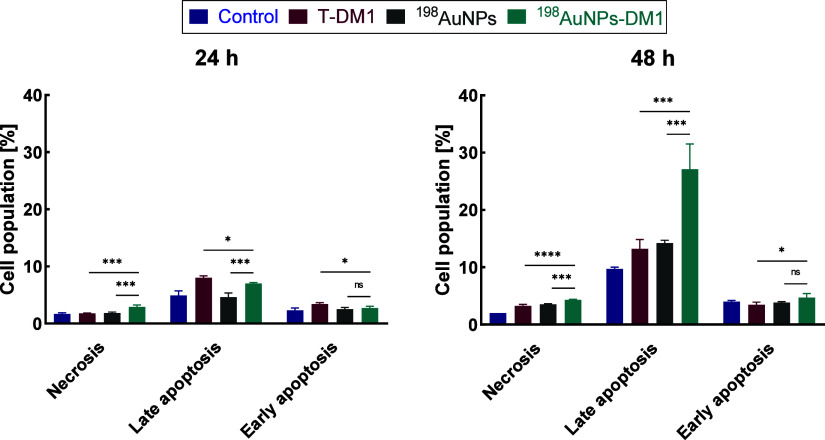
Apoptosis assay. Distribution of cell populations (necrosis, late and early apoptosis) treated with the T-DM1 (0.031 μg/mL), ^198^AuNPs (20 MBq/mL), and ^198^AuNPs-T-DM1 (20 MBq/mL; 0.031 μg/mL) compounds after 24 and 48 h. Untreated cells were used as control. A one-way ANOVA test was applied for statistical analysis; statistical significance was tested for T-DM1 vs ^198^AuNPs-T-DM1 and ^198^AuNPs vs ^198^AuNPs-T-DM1 groups. A *p*-value, *p* ≤0.05, was considered statistically significant (*), *p* ≤ 0.01 (**), *p* ≤ 0.001 (***), and *p* ≤ 0.0001 (****); nonsignificant (ns); mean ± SD, *n* = 3.

### 3.5 Spheroids

Drawing from the encouraging outcomes of apoptosis and MTS assays, the toxic effects of ^198^AuNPs,^198^AuNPs-T-DM1, and T-DM1 alone were evaluated using three-dimensional structures (spheroids). The 3D cell culture of spheroids displays functional heterogeneity,^34–36^ which includes cells residing in hypoxic environments and various phases of the cell cycle. This enables the study of cells with diverse proliferation statuses and heterogeneous responses to drugs. Moreover, studies^34,37^ have indicated that heterogeneous cell populations can include stem cells. For these reasons, 3D structures more accurately mimic the biophysical properties of tumors and the effects of drugs on them.^38–40^

In these experiments, one concentration of Kadcyla (0.031 ug/mL) and two doses of radiation (10, 20 MBq/mL) were tested. The study was conducted for 7 days until the tumor disintegrated (for the highest dose of combined therapy), and the results collected are presented in Figure 9. The findings are consistent with previous data. The strongest effect was observed for the highest dose (20 MBq/mL), with the greatest differences in spheroid area measurements noted after the third day of measurement. Control spheroids increased in the area, while treated spheroids decreased at varying rates. Spheroids treated with 20 MBq/mL decreased approximately 13-fold after 7 days compared to control spheroids (12 800 ± 300 μm^2^ vs 165 800 ± 600 μm^2^) and 9-fold compared to spheroids treated with T-DM1 alone (110 400 ± 200 μm^2^). Figure 10 includes microscopic images of the spheroids discussed. 3D cell cultures treated with radiobioconjugate decreased by almost 11-fold from day 0 (137 000 985 ± 900 μm^2^) to day 7 (dose 20 MBq/mL), while at the medium dose (10 MBq/mL) they decreased by more than 2.5-fold (137 000 ± 400 μm^2^ vs 52 00 ± 1600 μm^2^). A synergistic effect (*I* > 0) was achieved at the dose of 20 MBq, with an interaction index *I* of 0.415, whereas at 10 MBq/mL, *I* was 0.197.^30^ Palma Chaundler et al.'s work investigated the penetration dynamics of two ADCs, including Kadcyla, demonstrating that these compounds can fully penetrate spheroids within 24 h.^40^ Despite their large size, these biological molecules can induce similar toxic effects as smaller molecules. However, studies testing T-DM1 on spheroids showed higher IC_50_ values compared to 2D cell cultures, consistent with our results.

In Boyer et al.’s study, the effect of Kadcyla on several cell lines was investigated, revealing T-DM1 requires a longer incubation time for effective internalization in 3D spheroids or aggregates than 2D cultures.^34^ Kadcyla cannot efficiently penetrate dense and compact spheroids; initially, it primarily binds to HER2 receptors on the outer surface, where it undergoes internalization into the cells. As successive layers of spheroid cells undergo apoptosis, cells located inside become exposed to the compound. However, the situation differs when ^198^AuNP-T-DM1 interacts with spheroids. The extensive range of β^−^ particle interaction emitted by ^198^Au, up to 4 mm,^11^ allows the ^198^AuNP-T-DM1 conjugate attached to the surface to affect the entire spheroid.

**Figure 9:**
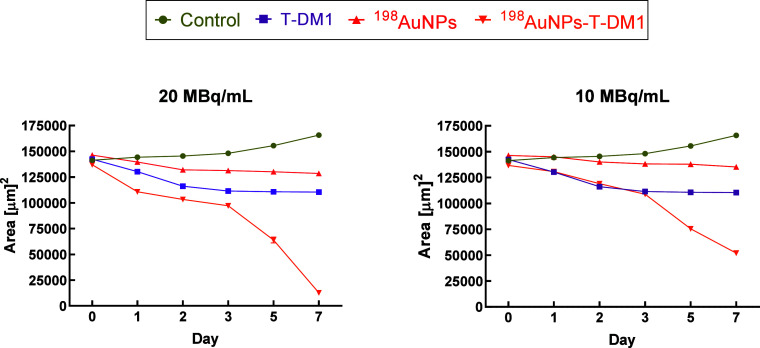
Time-dependent surface development characteristics of control SKOV-3 spheroids or after compound treatment: T-DM1 (0.031 μg/mL), ^198^AuNPs (10 and 20 MBq/mL), and ^198^AuNPs-T-DM1 (10 and 20 MBq/mL; 0.031 μg/mL). Untreated cells were used as control. Data represent the mean ± SD (*n* = 3).

**Figure 10:**
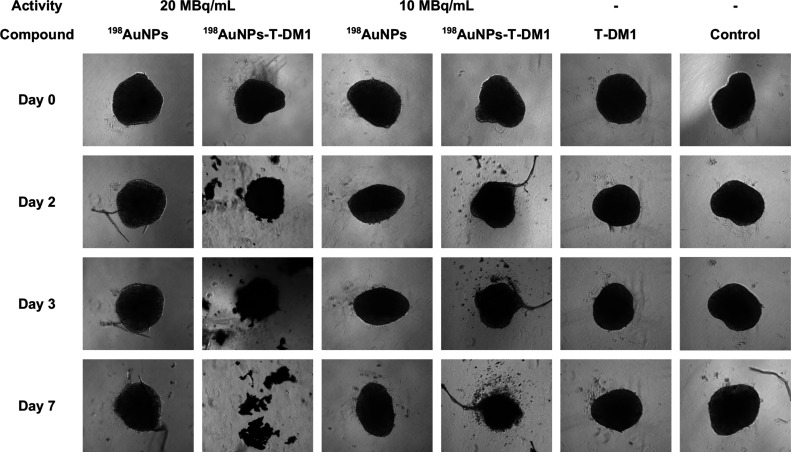
Microscopic images of the measured control and compound-treated SKOV-3 spheroids.

### 3.6 Cell Cycle Assay

To better characterize the mode of action of the radiobioconjugate, a cell cycle analysis was conducted using flow cytometry (Figure 11 and Figure S2). The greatest differences between phases were observed after 48 h. The analysis of the results was based on untreated control cells. Cells treated with radioactive nanoparticles were arrested in the G_2_/M phase, which correlates with their radiosensitivity in this phase.^41–43^ Cell cycle arrest in the G_2_/M phase was also observed in Kumar et al.’s study using another β^−^ emitting radionuclide, lutetium-177, where MG63 cells were treated with ^177^Lu-DOTMP.^44^ In the discussed experiment, the growth of cells in the G_2_/M phase was also induced by Kadcyla. This effect was more pronounced at 24 h (37.82 ± 0.92%) compared to the subsequent time point at 48 h (18.17 ± 0.55%). This finding is consistent with literature where BT-474 cells were treated with T-DM1 ^20^. Similarly, Montero et al.’s work examined the impact of Kadcyla on SKOV-3 cells, observing their accumulation in the G_2_/M phase of the cell cycle. The effect was most prominent after 24 h of incubation (at a dose of 50 nM, 7.425 μg/mL). Subsequently, the percentage of cells in the G_2_/M phase decreased over time, while the G_0_/G_1_ phase increased. However, the proportion of the G_0_/G_1_ phase compared to t_0_ was significantly smaller.^45^ In the current study, a lower dose of radiobioconjugate was used (almost 240 times lower), resulting in a lesser increase in cells in the G_2_/M phase.

The combination of trastuzumab emtansine and radiation, as expected, led to an increase in the number of cells arrested in the G_2_/M phase after 24 h, which was 36.88 ± 0.40%, and after 48 h, 37.51 ± 0.55%. Unexpectedly larger differences between measurement points were noted for the dose of radiobioconjugate 10 MBq/mL - 32.7 ± 2.0% (24 h), 46.9 ± 1.2% (48 h). This is a surprising and challenging effect to explain; however, the trend was maintained - an increase in the G_2_/M phase compared to control cells.

**Figure 11:**
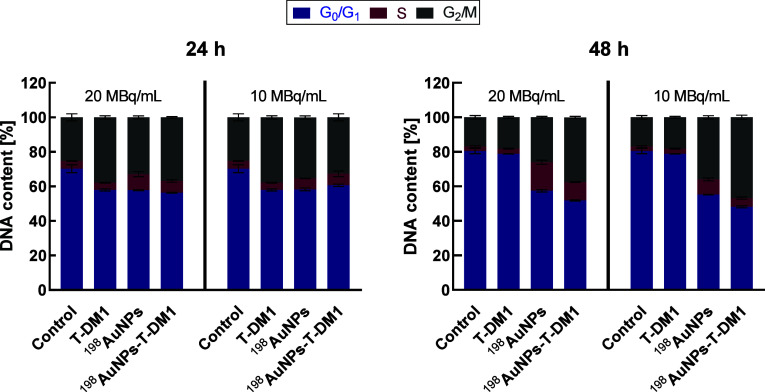
Cell cycle distribution of cell phases (G_0_/G_1_, S, and G_2_/M) after treatment with the following compounds: T-DM1 (0.124 μg/mL), ^198^AuNPs (10 and 20 MBq/mL), and ^198^AuNPs-T-DM1 (10 and 20 MBq/mL; 0.124 μg/mL) assessed at 24 and 48 h. Untreated cells were used as the control. Data represent the mean ± SD (*n* = 3).

## 4 Conclusions

This study proposes an innovative approach by combining ADC with radioactive gold nanoparticles aiming to enhance therapeutic efficacy. Unlike previous solutions that involved immobilizing chemotherapeutics, radionuclides and antibody-based vectors on nanostructured carriers,^9–11^ the ^198^AuNP-T-DM1 radiobioconjugate offers the significant advantage of releasing the chemotherapeutic agent after it reaches the therapeutic target. The released DM1 can then fully demonstrate its chemotherapeutic properties.

Optimal concentrations of potent T-DM1 were meticulously selected and validated for their successful attachment to nanoparticles. Several biological assays were conducted, beginning with the confirmation of T-DM1 binding intact HER2+ cell receptors. Encouraging results from subsequent internalization studies prompted further experimentation. Cytotoxicity studies provided evidence of synergistic effects at specific radiation doses (20 MBq/mL) combined with T-DM1. Treatment with 20 MBq/mL of radiation and Kadcyla concentration of 0.031 μg/mL resulted in spheroid disintegration within 7 days, underscoring the potential of this combined therapeutic approach. The findings suggest that integrating ADC therapy with radiation could potentially surpass the efficacy of standalone therapies, leveraging the strengths of each modality. The research presented in this study lays a robust foundation for future animal studies and potentially clinical trials in patients. Further exploration in preclinical models will be crucial to validate these promising findings and advance toward effective therapeutic strategies in cancer treatment. However, significant limitations must be considered regarding the use of this radiopharmaceutical. In vivo studies using the DOX-^198^AuNPs-Tmab radiobioconjugate in a murine tumor model,^9^ following intravenous administration of the compound, showed that it practically did not accumulate in the tumor but mainly accumulated in the spleen and liver. Conversely, direct administration of the radiopharmaceutical into the tumor resulted in nearly 100% retention at the target site—the tumor—without observed accumulation in other organs. Therefore, radiopharmaceuticals based on inorganic nanoparticles are dedicated to local administration directly into the tumor or into the postoperative cavity after its removal. It is important to note that classical labeling of Kadcyla, such as with ^177^Lu or ^225^Ac DOTA complexes, enables systemic administration of the radiopharmaceutical. However, the specific activity achieved (with a maximum of four radioactive atoms per trastuzumab molecule) does not substantially enhance the cytotoxicity of Kadcyla.

## Supplementary Material





## Notes

Summary of the interaction indices determined for ^198^AuNPs-T-DM1 responses in cytotoxicity studies, as well as flow cytometry plots of the cell cycle of SKOV-3 cells following treatment with the following compounds: T-DM1 (0.124 μg/mL), ^198^AuNPs (10 and 20 MBq/mL), and ^198^AuNPs-T-DM1 (10 and 20 MBq/mL; 0.124 μg/mL), assessed at 24 and 48 h (PDF)
