# Supplementary figures and images for: Radiobioconjugate of Kadcyla with Radioactive Gold Nanoparticles for Targeted Therapy of HER2-Overexpressing Cancers
Source: Mol Pharm. 2025 May 30;22(7):4019–31. doi: 10.1021/acs.molpharmaceut.5c00288 (PMC12239079; doi:10.1021/acs.molpharmaceut.5c00288)

Control

24 h

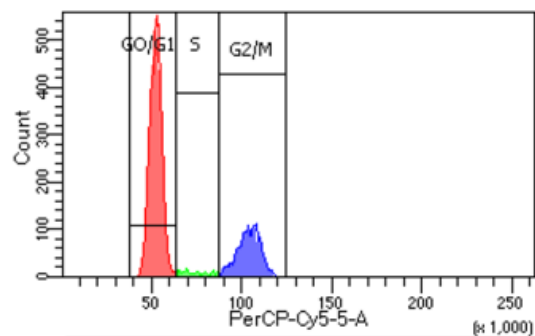

48 h

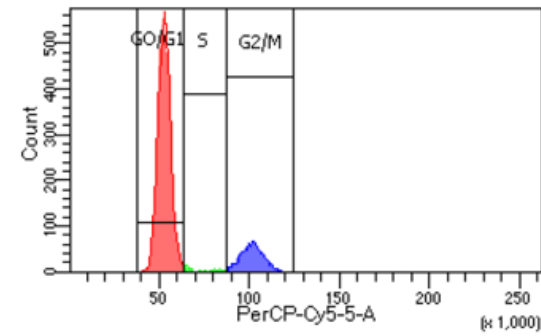

T-DM1

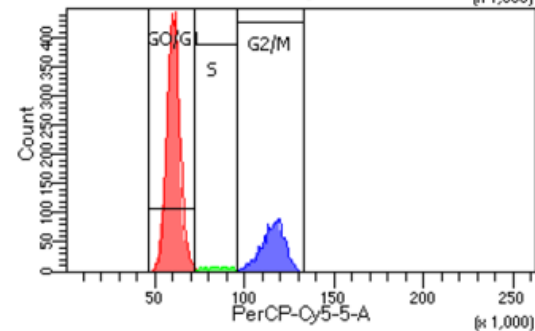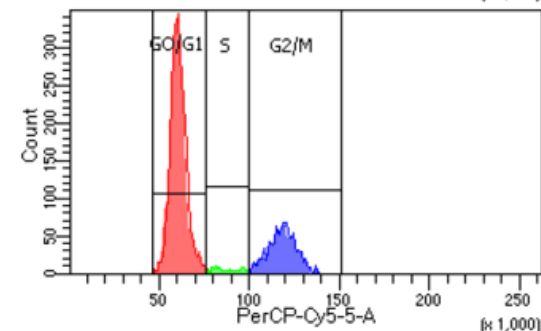

20 MBq/mL

10 MBq/mL

<sup>198</sup>AuNPs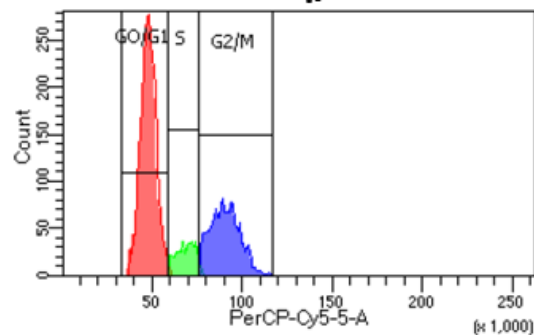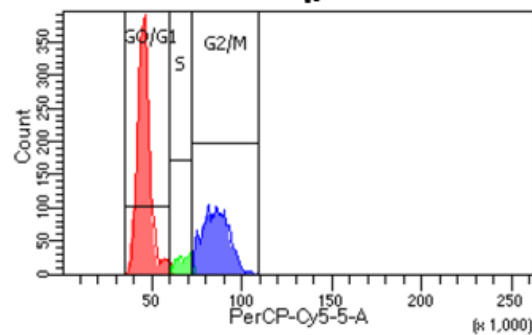

20 MBq/mL

10 MBq/mL

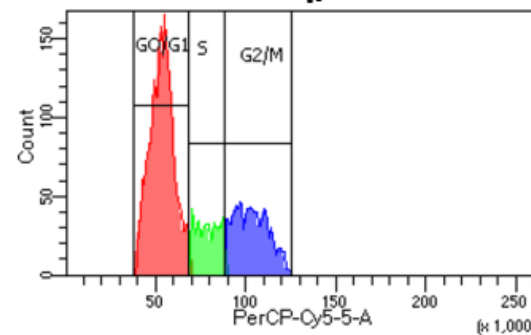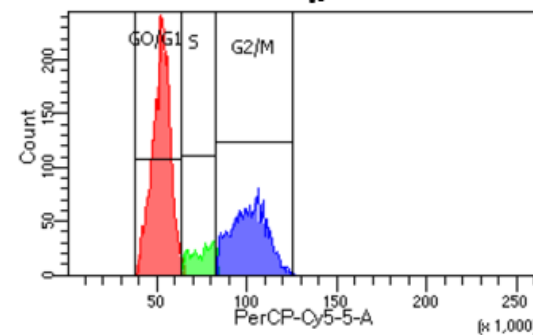<sup>198</sup>AuNPs-T-

DM1

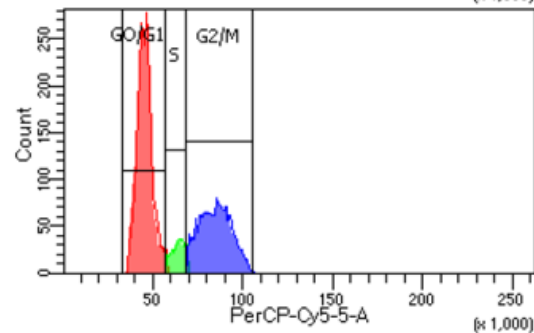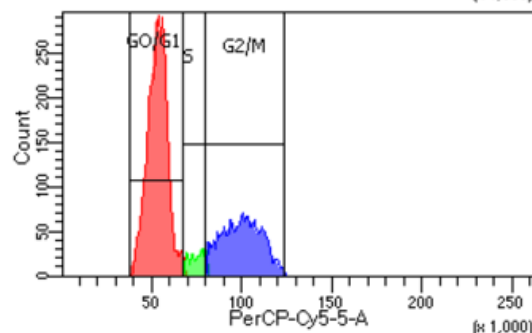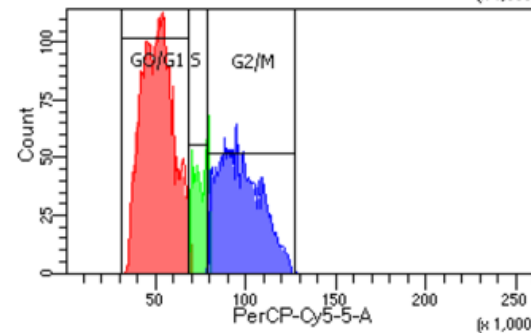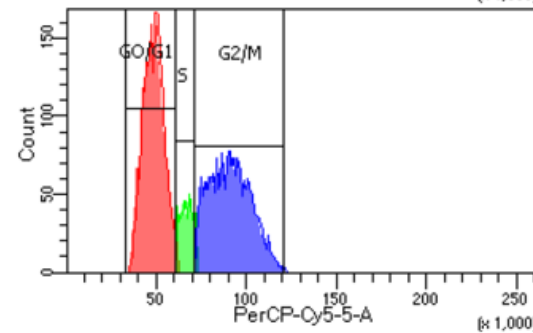

Supplement: Supplementary file 1 [file mp5c00288_si_001.pdf]
