# Supplementary material for: Radiobioconjugate of Kadcyla with Radioactive Gold Nanoparticles for Targeted Therapy of HER2-Overexpressing Cancers
Source: Mol Pharm. 2025 May 30;22(7):4019–31. doi: 10.1021/acs.molpharmaceut.5c00288 (PMC12239079; doi:10.1021/acs.molpharmaceut.5c00288)
Supplement: Supplementary file 2 [file mp5c00288_si_002.pdf]

Supporting Information

Radiobioconjugate of Kadcyla with radioactive gold nanoparticle for targeted therapy of HER2-overexpressing cancers

*Kinga Żelechowska-Matysiak[a], Kamil Wawrowicz[a], Mateusz Wierzbicki[b], Tadeusz Budlewski[c] Aleksander Bilewicz[a], Agnieszka Majkowska-Pilip[a,c]**

[a] Centre of Radiochemistry and Nuclear Chemistry, Institute of Nuclear Chemistry and Technology, Dorodna 16 St., 03-195 Warsaw, Poland
[b] Department of Nanobiotechnology, Institute of Biology, Warsaw University of Life Sciences, Ciszewskiego 8 St., 02-786 Warsaw, Poland
[c] Department of Radiology, Radiotherapy and Nuclear Medicine, National Medical Institute of the Ministry of the Interior and Administration, Wołoska 137 St., 02-507 Warsaw, Poland

**Correspondence: a.majkowska@ichtj.waw.pl*

Table S1. Summary of interaction indexes I determined for ^{198}AuNPs-T-DM1 responses in cytotoxicity studies.

**Study**	**Dose [MBq/ml]**	**Concentration T-DM1 [µg/ml]**	**Measurement point**	**Interaction indexes *I***	**Synergistic effect**
**MTS assay**	20	0.015	24 h	0.074	yes
			48 h	0.26	yes
			72 h	0.23	yes
		0.031	24 h	0.18	yes
			48 h	0.17	yes
			72 h	0.15	yes
		0.064	24 h	0.033	yes
			48 h	0.15	yes
			72 h	0.23	yes
		0.124	24 h	0.054	yes
			48 h	0.097	yes
			72 h	0.20	yes
	10	0.015	24 h	0.070	yes
			48 h	-0.071	no
			72 h	0.077	yes
		0.031	24 h	0.074	yes
			48 h	-0.14	no
			72 h	-0.012	no
		0.064	24 h	0.0071	yes
			48 h	-0.17	no

			72 h	0.017	yes
		0.124	24 h	0.057	yes
			48 h	-0.036	no
			72 h	0.15	yes
	2.5	0.015	24 h	0.048	yes
			48 h	-0.018	no
			72 h	-0.022	no
		0.031	24 h	0.13	yes
			48 h	-0.073	no
			72 h	-0.22	no
		0.064	24 h	-0.046	no
			48 h	0.039	yes
			72 h	0.021	yes
		0.124	24 h	0.072	yes
			48 h	0.056	yes
			72 h	0.10	yes
**Apoptosis**	20	0.031	24 h	-0.0085	no
			48 h	0.058	yes
**Spheroids**	20	0.031	7 days	0.42	yes
	10			0.20	yes

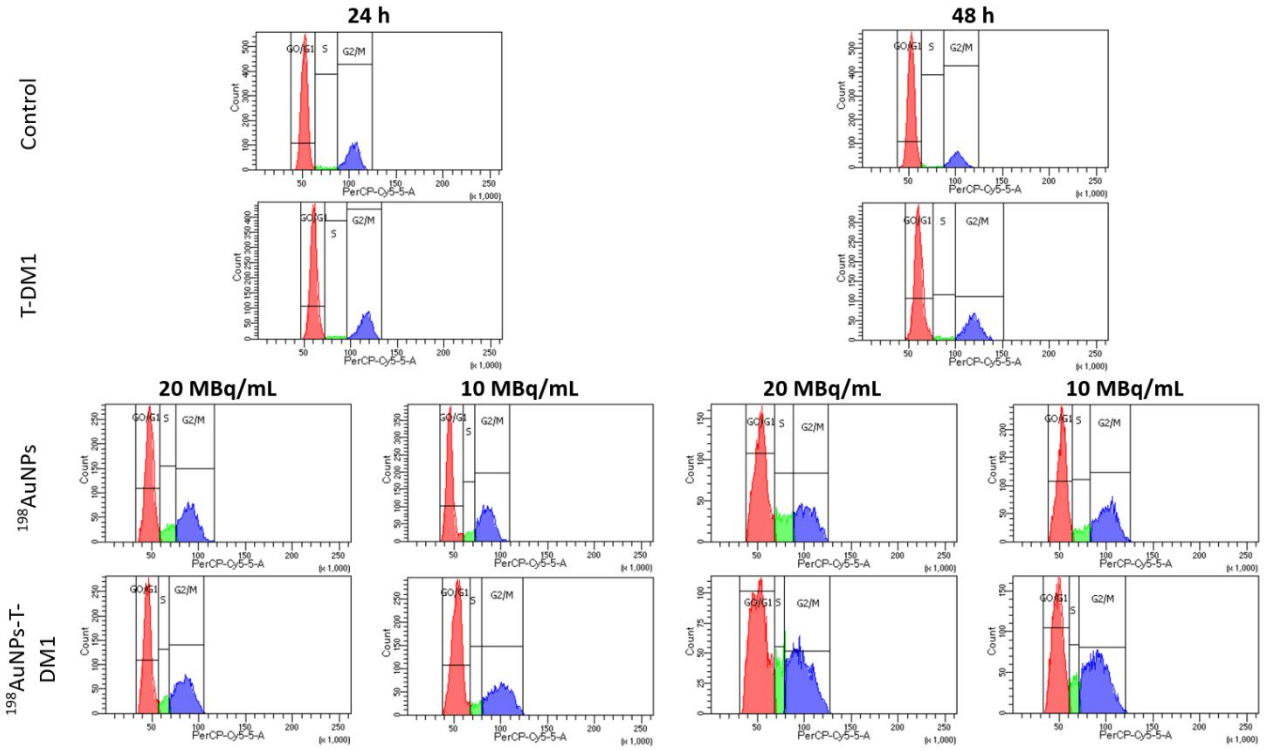

Figure S2. Flow cytometry plots of the cell cycle of SKOV-3 cells after treatment with the following compounds: T-DM1 (0.124 µg/mL), ^{198}AuNPs (10 and 20 MBq/mL) and ^{198}AuNPs-

T-DM1 (10 and 20 MBq/mL; 0.124 μg/mL) assessed at 24 and 48 h. Untreated cells were used as the control.
